# Extremely premature birth bioethical decision-making supported by dialogics and pragmatism

**DOI:** 10.1186/s12910-023-00887-z

**Published:** 2023-02-11

**Authors:** Joseph W. Kaempf, Gregory P. Moore

**Affiliations:** 1 Providence St. Vincent Medical Center, Women and Children’s Services, 9205 SW Barnes Road, Portland, OR 97225 USA; 2 Department of Obstetrics, Gynecology, and Newborn Care, The Ottawa Hospital – General Campus, 501 Smyth Road, Box 806, Ottawa, ON K1H 8L6 Canada

**Keywords:** Ethics-medical, Extreme premature birth, Decision making, Uncertainty, Value pluralism, Morality

## Abstract

Moral values in healthcare range widely between interest groups and are principally subjective. Disagreements diminish dialogue and marginalize alternative viewpoints. Extremely premature births exemplify how discord becomes unproductive when conflicts of interest, cultural misunderstanding, constrained evidence review, and peculiar hierarchy compete without the balance of objective standards of reason. Accepting uncertainty, distributing risk fairly, and humbly acknowledging therapeutic limits are honorable traits, not relativism, and especially crucial in our world of constrained resources. We think dialogics engender a mutual understanding that: i) transitions beliefs beyond bias, ii) moves conflict toward pragmatism (i.e., the truth of any position is verified by subsequent experience), and iii) recognizes value pluralism (i.e., human values are irreducibly diverse, conflicting, and ultimately incommensurable). This article provides a clear and useful Point-Counterpoint of extreme prematurity controversies, an objective neurodevelopmental outcomes table, and a dialogics exemplar to cultivate shared empathetic comprehension, not to create sides from which to choose. It is our goal to bridge the understanding gap within and between physicians and bioethicists. Dialogics accept competing relational interests as human nature, recognizing that ultimate solutions satisfactory to all are illusory, because every choice has downside. Nurturing a collective consciousness via dialogics and pragmatism is congenial to integrating objective evidence review and subjective moral-cultural sentiments, and is that rarest of ethical constructs, a means and an end.

**Supplementary Information:**

The online version contains supplementary material available at 10.1186/s12910-023-00887-z.

## Background

Accurately summarizing health outcomes and bioethical complexities related to extremely premature birth is challenging, perhaps unrealistic. The periviability literature is multiforme, lending itself to arbitrary selection of data and opinions of personal or institutional congruence, rather than broad consensus. Divergent cultural and religious beliefs, conflicts of interest, financial priorities, resource utilization, and the socio-economic struggles of families deserve far more scrutiny, yet persistently avoid analysis. Because of these peculiarities we submit an alternative model based upon the principles of dialogics and pragmatism. Our goal is to bridge the collective understanding gap within and between physicians and bioethicists.

Passionate ideology regarding palliative care versus neonatal intensive care for extremely premature infants hugely impacts health and social outcomes, but is curiously far less examined than medical interventions and therapies. Family preferences viewed as acceptable or not, sophisticated technologic therapies seen as experimental or not, and whether cultural heterogeneity is reducible to common moral foundations or not, are all issues beholden to disparate emotions and beliefs [1–4].

Physicians acknowledge uncertainty yet are risk averse. Arbitrary lines-of-demarcation that deny the ambiguous characteristics of nearly every feature of extremely premature birth tend to serve the interests of those with power. *Sorites Paradox* should be a feature of bioethical philosophy. When sharp demarcations cannot be established with assurance—“*This is clearly X and that is certainly Y*” – we are well-advised to minimize inflexible positions of “*X is right*” and “*Y is wrong*” [5–8].

Statements regarding what is moral imply what is not moral—the tautology that generates discord. The problem is that extreme prematurity disputes are scarcely ethical exercises of “*We agree to disagree*” because binary medical decisions are required in emergency scenarios. Without the luxury of time, key judgments must be made regarding fetal monitoring, antenatal corticosteroids, cesarean section, intubation, or not. Impending birth makes some decision paths convenient in the short-term for select participants, but later regretted and costly, depending upon subsequent events. We believe this *dissensus* is a fundamental of extreme prematurity, but not necessarily to be avoided nor solved, rather acknowledged and incorporated interactively along with features of *consensus* [9–13].

Because every choice has potential downside, extremely premature birth is a paradigm of human suffering and tragedy. There is no uniform pathway for anyone that eliminates risk, pain, and guarantees well-being. The decision-making process and family reactions to clinical events ebb and flow with changing notions of “*right*” and “*wrong*”. Physicians should avoid framing extreme prematurity and periviability choices as an opportunity for pregnant women and families (and least of all physicians) to be noble or heroic [14, 15].

Our goals: i) capsulize recent extremely premature infant long-term neurodevelopmental outcome reports to bolster objective shared decision-making (Additional file 1: Table S1), ii) provide a practical Point-Counterpoint of divergent viewpoints related to extreme prematurity (Table 1), and iii) illustrate the productive nature of dialogics and pragmatism as the methodologies that encourage listening and information exchange, and the relational interactions that nurture a collective consciousness which enhance empathetic understanding and bioethical decision making. Readers are encouraged to reflect upon this manuscript because understanding the nature of dialogics and pragmatism will encourage their important contributions to our understanding of extreme prematurity.

**Table 1:** Point—Counterpoint dialogic summary of the principal viewpoints and issues of extremely premature birth—Neonatal Intensive Care versus Palliative Care

Neonatal intensive care for extremely premature infants	Palliative care for extremely premature infants
Gestational age estimates are imprecise, ± 1 week, the 23 week infant might be 24 weeks	Gestational age estimates are imprecise, ± 1 week, the 24 week infant might be 23 weeks
Gestational age is the most common prognosticator of mortality and morbidity but can be imprecise and is not the sole determinant of outcomes	Birth weight correlates with mortality and NDI as effectively as gestational age, and ultrasound can reasonably estimate fetal weight. Sex, multiple gestation, fetal anatomic survey, maternal biomarkers, medical, and demographic descriptors also augment prognostication
Guidelines based upon gestational age are biologically artificial. It is arbitrary to recommend intensive care at 24 0/7 weeks but palliative care at 23 6/7 weeks	All published guidelines use gestational age as a framework. Inflexible gestational age line-of-demarcation arguments resist logic. It is arbitrary to recommend palliative care at 21 6/7 weeks but provide NICU care at 22 0/7 weeks
Survival is a problematic forecast in NICUs that promote palliative care, a self-fulfilling prophecy of mortality	Shared decision-making and family preference are compromised in hospitals that mandate NICU care. This is a self-fulfilling prophecy of pain, suffering, morbidity, late hospital mortality, and NDI
All EPIs should be resuscitated and re-evaluated daily; life support withdrawal is morally acceptable only after a “trial of life” which might improve prognostication	EPIs with late hospital mortality or subsequent NDI do not consistently have early, severe morbidities that prompt stopping life support. Withdrawing life support later in the course can contradict the rationale for the initial intensive care, this is ethically inconsistent
EPIs can survive and be healthy, this is difficult to determine in the delivery room or first days of life	EPIs can appear stable in the delivery room or first days of life but then suffer major morbidities, late mortality, and/or NDI. This prediction is difficult to make accurately in the delivery room or first days of life
NICU outcomes presented as percentages can be poorly understood by families and might be misleading if biased by information-framing	Every authoritative consensus statement recommends shared decision-making enhanced by evidence-based short and long-term outcomes review. This requires practical use of numbers, proportions, and percentages presented clearly
Medical science cannot advance unless therapeutic frontiers are pushed. Withholding possibly beneficial technology is coercive and restrictive, a power differential that risks abuse	Pregnant women and families have the right to decline unproven, or high-risk maternal and NICU care therapies because of the subsequent pain, late mortality, NDI, chronic health issues, and other unforeseen consequences
Survival of EPIs has improved over time and might continue if we keep trying	Detailed descriptions of pain, and NICU or post-discharge deaths are inadequately described in publications. How much suffering and how many deaths justify unproven, experimental therapies is not a consensus agreement among physicians, families, and society
Withholding and withdrawing life support are morally equivalent	Withholding and withdrawing life support are moral equivalence theories, but are not ethically equivalent realities to all families or providers. Physicians and families recognize when early institution of palliative care minimizes unnecessary pain, suffering, and moral distress
The core issue: do we have a right or defensible argument to deny a trial of life support to an EPI, or any sentient human?	The core issue: is a pregnant woman, because she may deliver an EPI through no choice of her own, morally required to assent to NICU care regardless of family circumstance, preferences, risks to her well-being, and uncertain, suboptimal pediatric long-term health and neurodevelopment?
A principal challenge with authentic shared decision-making is minimizing physician bias	A principal challenge with authentic shared decision-making is ensuring families know their informed decision must often be made in a time-sensitive fashion, and that no decision is a decision
All human life is sacred, God should play the principal role in deciding which person lives and who dies. Medical therapy is an extension of supernatural power	Sacred is a religious concept not shared by all families and physicians, who differ by creed or culture, or might be non-theists. All human beings are not viewed as “persons” with equivalent rights by every family. This distinction is made with major birth defects where there is broad agreement of limitations of NICU therapies
How can the “best interests of an infant” ever be death? Unless early death is near certain, NICU care should be attempted	Death versus pain, suffering, and significant NDI are incommensurable outcomes, reflective of value pluralism inherent to the human condition and a continual source of conflict. “Best interests of the woman, family, and infant” is a more realistic, inclusive consideration
Palliative care can be perceived as giving up, might have a variable course, and be associated with subsequent family regret	Palliative care can be delivered as well-planned support, a humane process of dignity, family advocacy, and the reduction of pain and suffering. NICU care, pain, late mortality, and NDI can be associated with subsequent family regret
Some countries do not permit therapeutic abortion at gestational ages that EPIs receive intensive care, so palliative care is inconsistent with these laws	Some countries do permit therapeutic abortion at gestational ages that EPIs receive intensive care, so mandating intensive care is inconsistent with these laws
Palliative care leads to the death of infants without giving them a chance. They could possibly be healthy children, or with chronic medical conditions yet rate their lives as good	Mandated NICU care is of little personal risk to physicians. Surviving EPIs may be chronically unhealthy. It is the families who bear the risks, the principal costs, the disruption, and it is EPIs who physically suffer
Religious families may prefer NICU care for their infant, for reasons not necessarily evidence-based. We generally respect their sentiments and rights. Variability exists both between and within many faith traditions	Religious families may prefer palliative care for their infant, for reasons not necessarily evidence-based. We generally respect their sentiments and rights. Variability exists both between and within many faith traditions
Women who have extra-ordinary pregnancy circumstances, advanced maternal age, infertility, or serious medical conditions should be supported if they desire NICU care for their EPI	Women who have extra-ordinary pregnancy circumstances, advanced maternal age, infertility, or serious medical conditions should not be expected to desire nor choose NICU care for their EPI
Wrongful EPI death lawsuits have been litigated and settled	Wrongful EPI life lawsuits have been litigated and settled
Physicians and bioethicists who advocate palliative care options might represent a minority position within NICU care proponents, especially among neonatologists	Physicians and bioethicists who are NICU care proponents emphasize their disagreements with palliative care advocates, rather than acknowledging that their fundamental disagreements are with pregnant women and families who choose palliative care
Physicians who favor palliative care may be influenced by lack of strong interest in EPI care, fixed salary structure regardless of census, inferior bedside skills, burnout, religious and cultural beliefs not shared by families, or lack of compassion for those with NDI	Physicians who favor NICU care may be influenced by prestige, research interests, career advancement dependent upon EPI care, financial incentives based upon census, religious and cultural beliefs not shared by families, and attraction to hero-victim relationships
Physicians who have children or are parents of EPIs might have unique insights, and their shared experience can be relevant to other providers, pregnant women, and families	Insight, which some physicians might have based upon their own child or EPI, is distinct from moral authority, of which physicians have no more credibility than others. Physicians must be vigilant to not imply they have ethical prowess whether they have children or not
The death of an EPI is not morally less consequential than the death of a ten-year-old, or a forty-year-old. Palliative care devalues EPI lives as compared to older children who would receive intensive care for comparable conditions	Judgments about meaning or morality of EPI deaths as compared to deaths of older children or adults are incommensurable value judgments, culturally divergent, and not necessarily shared by those families that have experienced one or the other, or both
The cost of EPI care is proportionately small compared to what is spent on adults with similar or even worse prognoses. Acceptable quality years of life can result from EPI intensive care as compared to adult intensive care	Resources required for EPI care are diverted from other healthcare needs that are more cost-effective for women and children, which diminishes population health. Financial burdens to families for long-term EPI care is enormous, and poorly supported. The lost opportunity costs for families are seldom considered
Adults with critical illnesses that have similar risks of mortality and morbidity as EPI conditions might receive intensive care without controversy. It is unjust to treat EPIs differently	Adults with critical illnesses that have similar risks of mortality and morbidity as EPIs are allowed (or designated surrogates) to choose palliative care without controversy
Many surviving EPIs do not have “Significant NDI”, this justifies broad application of intensive care. If approximately 35–45% of surviving EPIs have significant NDI this implies 55–65% do not	“Significant NDI” is defined as a composite cognitive and motor assessment > 1–2 SD below the mean. A large portion of surviving EPIs have NDI complicated by a broad spectrum of neurobehavioral and psychiatric challenges. There is no significant secular time trend of improvement in NDI in 22–24 week infants in the past 30 years
Some adolescents and adults with NDI rate their quality of life highly and have similar achievement levels and social functioning as term infants	Some adolescents and adults with NDI rate their quality of life significantly lower than those persons born at term. Parents generally rate EPI quality of life lower with time. Quality of life assessments are unobtainable from persons with severe NDI
Persons with NDI should not be judged as less valuable than those without NDI. It is arbitrary to decide what type of neurologic functioning is “normal” versus “handicapped”	Brain injury prevention is an unequivocal research and quality improvement priority. NDI is widely accepted as undesirable. NDI is not a “neurodiversity” condition pregnant women or families would choose for their child
Palliative care advocates may not demonstrate compassion for EPIs or their families. Lack of empathy can lead to short-term convenience decisions, even apathy. Lack of sympathetic caring perpetuates nihilism	Intensive care advocates conflate compassion and pity. Pity enhances physicians’ status by reducing families to neediness. Physicians should not create meaning for themselves by using power over those they designate as needing help. This perpetuates nihilism
Uncertainty means we do not know what will happen and is typically the physician’s focus. Ambivalence and ambiguity can generate intensive care as the default recommendation	Risk is the product of something harmful occurring multiplied by the probability it will happen (which incorporates uncertainty). This is the pregnant woman and families’ focus. Ambivalence and ambiguity can generate palliative care preference
Some physicians and ethicists promote the descriptor “Gray Zone”, an undefined, but assumed shrinking estimate of extreme prematurity. This time period is characterized by NICU outcomes being so uncertain and risky that family choice of palliative care or NICU care is reasonable	Some physicians and ethicists prefer the descriptor “Zone of Parental Discretion”, a variably-sized-by-culture, ethically-protected-by-circumstance, time period of extreme prematurity. NICU outcomes are so uncertain and risky that family choice of palliative care or NICU care is reasonable

*Dialectics* (thesis-antithesis-synthesis) assume there is inherent, constant progress toward some inexorable goal or hallowed truth, and are especially seductive in healthcare where absolutism, technology, and scientism are dominant motifs. Absolutism requires participants acquiesce to the cultural, religious, or political beliefs of others in power, and scientism is believing the scientific method and technology are the ultimate paradigms for solving human problems [16–18]. In sharp contrast, *dialogics* highlight that ultimate, ordained solutions to complex ethical issues have never in human history been entirely rational, objective, much less satisfactory to all [19–21].

*Dialogics* are consummately suitable to extremely premature births because this communication philosophy recognizes that language and information exchange affect us in multiple directions that resist convenient synopsis. We modify words and data (and vice versa) as continual interactions and flux, each of us biased, culture-influenced, yet not necessarily fixed in sentiment or position [18–20, 22, 23].

Moral intuitions are neither fixed deontological maxims nor calculable utilitarian equations, but rather rooted in biologic, social, and ideologic underpinnings that are products of human evolution [24]. These intuitions, if part of reflective *dialogic* equilibrium, become part of our collective consciousness [25]. The present affects our evaluation of the past no less than the reverse. Each person’s beliefs and authority in evolving *dialogics* hold more, or less, salience and authenticity depending upon the circumstance and precise issue, and not as hierarchy, but as interactive network dynamics [26].

*Dialogics* shun absolutism, and incorporate value pluralism rather than relativism, a crucial distinction. Value pluralism: *History consistently illustrates that core human values conflict, are often incommensurable with no common currency, may or may not be rational, and are inherently irresolvable absent of hierarchy and power differentials.* In contrast, moral relativism: *You think two situations or judgments are morally different, but you are mistaken, they are morally equivalent* [16, 17, 27]. *Dialogics* bolster pragmatism, i.e., the useful truth of any belief or policy is steadily borne out by the sum of all subsequent events. Pragmatism is empiric validity, not compromise; it is instrumental to fact-finding, not mere opinions devoid of objectivity and principle [28].

## Main text (dialogic discussion)

**Author A**: Anxiety can spring from denial of self-responsibility. Our lives possess accountable freedom to create authenticity without suppliance to external authority or supernaturalism. Within extreme prematurity conflicts we disguise angst by drifting to unrestrained interventionism and technology (scientism). When we avoid the self-responsibility of deciding collaboratively what is truly meaningful as a community in the here-and-now, then we perpetuate healthcare dysfunction, even nihilism [29].

**Author B**: Decisions for extremely premature infants are life-altering, families should not be denied the opportunity of self-responsibility for decision-making [30]. Nor should appeal to external authority or supernaturalism be rebuffed. Supernaturalism provides a foundation for some families, allowing them to cope with the uncertainty of life-and-death decisions [31]. Denying religious values could drive nihilism. Yet supernaturalism—or any belief system—can result in care requests many physicians consider morally wrong.

**Author A**: Physicians confuse compassion and pity. Compassion is sympathetic concern for others’ suffering and is innate to human nature. Pity enhances oneself by reducing others to neediness. We create meaning by being powerful enough to help. Pity falsely stages us as better than others, this perpetuates dependence and vulnerability [32, 33].

**Author B**: Rather than differentiating compassion and pity, we should emphasize to pregnant women and families *“I imagine you would prefer to not be in this difficult position, but you are, and I am here to inform and support you.”* This does not imply *“It is difficult for you to decide what to do, so let me decide.”* Communicating with compassion the truth of unbiased, hopeful, frightening information regarding risks and benefits of care options is essential to informed consent [34].

**Author A:** Social media influencers often imply honesty is the principal issue of conflict. But it is not honesty that is the modern arbiter of disputes, it has become sincerity. Sincerity is conflated with honesty. If I hold a sentiment deeply for my special reasons, it becomes inviolable. This has become preferable to dialogics and pragmatism; it is me claiming my rights. In periviability controversies, physicians convey sincerity as part of their authority; we can be sincere but not honest, a substitute for integrity [35].

**Author B:** Fundamental to periviability dialogics is tolerance. Replies to value-laden questions, e.g. “*What would you do doctor?”,* are acceptable assuming declaration of physician values and biases accompany the reply [36]. A sincere answer that involves non-disclosure of evidence-based information inherent to authentic dialogics is regrettable. Examples of intolerable physician answers might be *“There is unfortunately nothing we can do at 22 weeks”* or *“Babies born at 25 weeks do well, so intensive care is best.”* Physicians should acknowledge the way evidence is presented can be either positive (“survival”, “free of neurodevelopmental impairment”), or negative (“death”, “neurodevelopmental impairment”), or honest (providing both the positive and negative framing of evidence) (Additional file 1: Table S1).

**Author A:** We respectfully accept pregnant women discussing their religious values. But physicians should not assume pregnant women nor colleagues desire to know practitioners’ religious beliefs, much less agree with them. Inserting religious or political doctrine is particularly egregious with pregnant women because of their vulnerability. They deserve sympathetic understanding and medical expertise, not personal bias and absolutism. Deeply religious individuals of various faiths choose palliative care in similar scenarios that other equally devout people choose intensive care. Physicians may have difficulty integrating this characteristic of value pluralism [24, 37].

**Author B**: Most people have a worldview that guides decision-making. A physician’s dialogic and pragmatic role is not primarily to make the decision, but to clarify, guide, and support compassionate, reasoned pathways. If physicians are clear regarding their function, then beliefs that they legitimately possess should have minimal effect on the pregnant woman’s decision [38, 39]. Physicians should understand that whether particular neurodevelopmental impairment(s) are “significant” in the real world varies among institutions, countries, and between practitioners and families based on qualitative judgements. There are no precise, universal normative categories regarding the quality of life or value of children.

**Author A:** Ambiguity encourages wishful thinking, which leads to “*right*” and “*wrong*” declarations. This devolves to “*I think this is good, you should too*.” Physicians create tragedy because lines-of-demarcation in ethical controversies are seldom logically defined nor clinically absolute (*Sorites Paradox*). Lines-of-demarcation declared definitive often originate from hierarchy and power-differentials [27, 40, 41].

**Author B:** Practicality necessitates certain lines-of-demarcation. A simple analogy is the selection of voting age in democracies. If it is age 18 years, then some individuals 17 years + 364 days could surely make an educated choice but are not allowed, simply to facilitate clear, implementable voting processes. When there is a spectrum of moral opinions within a group of healthcare providers, choosing a line-of-demarcation can allow a coherent, implementable team approach. The choice of such a line should not be permanent. The bioethical culture of healthcare should adapt when sociological, epistemological, or medical findings necessitate change [42].

**Author A:** Physicians advocate evidence-based medicine in general, yet inconsistently in periviability decisions. Standardized, informed consent is arbitrarily applied, e.g., recommended for surgery but not broader life support interventions [43, 44]. Extreme prematurity is laden with uncertainty, risk, and experimental therapies. Neonatologists should not assume specialized knowledge of medical conditions legitimizes their moral authority. Evidence based practices and compassion are family-centered bedrocks that begin before decisions regarding extremely premature birth are made, not after admission to the NICU [11, 12].

**Author B:** Sound arguments support the importance of structured, informed consent. The disclosure of relevant knowledge is foundational to the consent process. Understanding information enhances the autonomy of a family. We accept pregnant women as surrogate decision-makers and support them by appropriate disclosure of information [45]. The volume and technical content can overwhelm decision-making capacity. Studies of decision support tools suggest parents think the amount of information as enough, and if anything, be increased. Under-informing pregnant women is a greater impediment to informed consent than over-informing [46, 47].

**Author A:** Survival rates are increasing but neurodevelopmental impairment rates, particularly < 25 weeks gestation, are not improving [48]. In fact, outcomes may be worsening over time when broader neuropsychiatric conditions are included (Additional file 1: Table S1). Quality of life is rated lower in surviving extremely premature infants as they become teen-agers and adults [49–52]. Advocates of universal intensive care conflate improving survival and not-improving long-term health. This enables their authority of ever-expanding intervention, despite publishing no evidence of improving comprehensive neurodevelopmental outcomes [53, 54]. Claims of conscience are valid bidirectionally: “*I choose not to participate in palliative care*” and “*I choose not to participate in intensive care*” are both legitimate. Physicians who support palliative care options no more find “*favor*” with an infant death than a physicist finds “*favor*” with gravity [55, 56].

**Author B:** Fear of death is a universal apprehension; for physicians this includes the fear of causing death. Given the inevitable result of palliative care, we may possess a heuristic that predisposes toward advocating intensive care. Introspection about death and the moral status of the fetus/newborn challenges us to study broader empirical experiences of women and families of diverse culture and circumstance who have lived all the different care choices, and thus understand multiforme long-term outcomes [57, 58]. Gaining more empirical data about how physicians may experience fear in terms of “causing death” or “creating children with neurodevelopmental problems” would advance our understanding of physician normative views.

**Author A:** Certain population healthcare objectives are unambiguous—providers receive ‘X’ dollars to care for ‘Y’ patients per annum. This requires priority-setting, inexorable difficult choices, all with downside [59, 60]. If we spend $1000 here, we do not spend $1000 there. We make these choices outside healthcare as a matter of routine, e.g., household expenditures, public schools, city infrastructure, social supports. Neonatology is not exempt from evidence-based, population health priority-setting [61, 62].

**Author B**: Physicians are not economists. We should be cognizant of resource utilization, but our proper focus is caring for the patients in our presence with the resources at our disposal. Peter Singer’s “*drowning child*” analogy is pertinent. We would all save a nearby child, even if it meant ruining expensive shoes by stepping into water, yet we might neglect a child dying from famine in a distant part of the world.

**Author A**: Health equity is social justice, an aspiration more vogue than reality. In the United States, we rationalize organ transplants in senior citizens, expensive genetic treatments for rare disorders, and high six-figure dollar expenditures for extremely premature infants, yet require families to pay for prenatal and well child visits, routine birth care, immunizations, effective medications, and emergency care. Extreme prematurity’s peculiar admixture of unproven interventions, unrestrained technology, uncertain outcomes, and disruptive financial costs consistently imperil family well-being, thus palliative care should not be oversimplified as cost-cutting [62–66].

**Author B:** Infant mortality in Canada was 4.4 per 1000 live births in 2019. The rate was 16.7 in the territory Nunavut compared to 4.5 in the province Ontario [67]. This discrepancy objectifies healthcare inequity, is unchanged over the past 20 years, and suggests injustice. Every human life has value and the rising survival rates of extremely preterm infants over this same 20 years is considered testimonial (Additional file 1: Table S1). How do we reconcile improved survival of extremely preterm infants amidst widely discrepant subpopulation rates?

**Author A:** Extremely premature birth is a significant health risk for pregnant women [68, 69]. Cesarean section rates are reported as 31% and 69% in 22–23 week and 24–25 week infants respectively [54]. Obstetricians’ fundamental duty is to protect the autonomy and well-being of pregnant women first-and-foremost, and to avoid unreasonable health risks thrust upon them by neonatal intensive care.

**Author B:** Protecting the autonomy and health of our patients is a given. If the pregnant woman is not healthy, the fetus will be adversely affected. Studies regarding routine cesarean section in extreme prematurity do not provide risk–benefit clarity, complicating uncertainty about providing palliative care or intensive care for the infant [70]. Shared decision-making should be used to support the pregnant woman regarding cesarean section decisions if intensive care is going to be provided to the infant [11, 12, 30, 71].

**Author A:** Because the majority of 22–23 week infants and many 24 week infants who receive intensive care either die in the neonatal intensive care unit (NICU) or survive with significant neurodevelopmental impairment and chronic health problems (Additional file 1: Table S1), NICUs and physicians who actively promote or mandate care at these extremes should do so without charges to insurers, the government, or families, whatever the outcome. This would add credibility to programs that describe their model as “*proactive*” or a “*positive approach”* [53, 54].

**Author B:** Is it eugenic to deny life-sustaining therapy to extremely premature infants at a certain gestational age? Choosing to provide care only to select patients without accepting that one may be wrong and causing harm by this choice carries an undesired sense of infallibility. Framing palliative care as negative versus intensive care as positive is mistaken. It is incongruous with the expanding availability of medical assistance in dying, choosing death in the face of suffering and impairment. Is there justification for pregnant women to exercise surrogate authority to allow their extremely preterm infant to die while some societies also allow autonomous adults to choose euthanasia? Do we have enough certainty about “*best interests*” for each extremely preterm infant such that we should override this surrogate authority?

**Author A:** NICUs with high rates of palliative care or conversely intensive care for 22–24 weeks infants should be transparent if and how authentic shared decision-making occurs. This is unequivocally recommended by authoritative consensus statements [12, 30, 71]. Hospitals that do not provide palliative care, or alternatively intensive care, at 22–24 weeks should offer the option of safe transfer of the pregnant woman to a hospital that honors her well-being and informed choice.

**Author B:** The difficulty with high-risk care is some pregnant women cannot safely be transferred because of unstable clinical factors. These women will potentially deliver at a hospital that cannot necessarily honor informed choice, or might be unable to provide the chosen option for certain clinical conditions due to expertise, staffing, and/or equipment. Undesirable mortality and morbidity might result.

**Author A:** Physicians are no more rational nor impartial than the general citizenry. We warrant the same scrutiny as politicians, scientists, philosophers, educators, et cetera. Humans like something and call it *“good”*, not the opposite [24, 25, 27]. Physicians and bioethicists should recognize there are no objective, ideal, nor supernatural “truths” existent in Nature, religion, science, or philosophy to guide ethical behavior [16, 18, 21]. Civilized society does its collective best by using pragmatic means of reason, justice, compassion, and dialogics [16, 19, 20]. Scientism and technology should not provide cover for hierarchical, unreasonable ideologies. We regrettably disguise our biased *prescriptions* as *descriptions*, cloaking our anxiety and uncertainty with opinion disguised as special facts that we alone understand [37, 72, 73].

**Author B:** Ideally, equipoise characterizes care options for extremely preterm infants—some view intensive care as an uncontrolled experiment, others view palliative care as discriminatory [74–76] (Table 1). If we provide pregnant women the opportunity to participate in comprehensive, longitudinal research studying decision-making, long-term health outcomes, and family sentiments then we might overcome inherent biases, and promote compassion and justice. We particularly need inquiry regarding women and families whose infants received palliative care, and how this compares to those whose infant received intensive care.

## Conclusion

Suffering is the universal constant of human experience. How and why we minimize needless anguish yet nurture our will-to-flourish is paramount in a civilized society [16, 77]. Humanity draws inspiration from diverse philosophies, religions, science, literature, quantum physics, and more. We believe these all authenticate the singular wisdom of *dialogics*—the cosmopolitan ability to communicate, learn, and adjust that does not necessitate immediate consensus nor solutions [78–80].

We have highlighted respectful pragmatism and value pluralistic mind-sets of well-being that are the subsequence of interactive relationships. The purpose is to nurture a more collective consciousness, rather than dogma or mythical objective truths, within and between bioethicists and physicians. *Dialogics* incorporate suffering as unavoidable, but also as a foundation for living with extremely premature birth controversies. Human will-to-flourish is an individual impetus and yet a communal opportunity to grow, recognizing we are inherently social beings. *Dialogics* is that rarest of ethical constructs – both a means and an end, categorically of vital importance.

## Supplementary Information

**Additional file 1**: **Table S1**. 2016–2022 summary of recent neurodevelopmental follow-up studies of extremely premature infants.

## Data Availability

All data generated or analyzed during this study are included in this published article.

## Abbreviation

NICU: Neonatal intensive care unit
