# Supplementary material for: Extremely premature birth bioethical decision-making supported by dialogics and pragmatism
Source: BMC Med Ethics. 2023 Feb 11;24:9. doi: 10.1186/s12910-023-00887-z (PMC9922460; doi:10.1186/s12910-023-00887-z)
Supplement: Supplementary file 1 — Additional file 1: Table S1. 2016–2022 summary of recent neurodevelopmental follow-up studies of extremely premature infants. [file 12910_2023_887_MOESM1_ESM.docx]

**Supplemental Table.** Summary of recent neurodevelopmental follow-up studies of extremely premature infants.

| **First Author, Journal, Year,**  **PubMed Identification Number** | **Report Summary: Assessment age; Assessment of; Birth years** | **Population:**  **Gestational Age or Birthweight** | **Locale** | **Principal Findings** |
| --- | --- | --- | --- | --- |
| Joseph, *Pediatrics*, 2016, PMID 27006473 | Assessment at 10 years; IQ, cognitive and academic; 2002-2004 | 23-27 weeks GA | USA, ELGAN cohort | 33-66% of survivors performed >1 SD below age on validated cognitive and academic tests, with the most extensive impairments at 23-24 weeks GA. |
| Serenius, *JAMA Pediatrics*, 2016,  PMID 27479919 | Assessment at 2.5 and 6.5 years; NDI; 2004-2007 | 22-26 weeks GA | Sweden, EXPRESS cohort | 36% of survivors free of NDI. 47% remained in the same NDI category at 6.5 years, 21% moved to a better, 32% to a worse category. 96%, 79%, and 63% of infants born at 22, 23, or 24 weeks either died or had moderate-or-severe NDI. |
| Cheong, *Pediatrics*, 2017, PMID 28814550 | Assessment at 8 years; IQ, academic, neurosensory;  1991-92 vs. 1997 vs. 2005 | <28 weeks GA | Australia,  Victoria cohorts | Major neurosensory impairment rates (15-18% of survivors) have not changed across 3 eras while problems in academic performance increased in the latest cohort (14-23% in 2005 vs. 5-10% in 1997). |
| Holsti, *Pediatrics*, 2017, PMID 28108580 | Assessment at 10-15 years; chronic conditions, IQ, motor;  1992-1998 | 23-25 weeks GA | Sweden, two centers,  see also Samuelsson below | Significantly higher functional limitations (64% vs 6%), service needs (64% vs 25%), CP (9% vs 0%), and IQ >2 SD below the mean (31% vs 5%) in adolescents born preterm compared to term controls. |
| Mathewson, *Psychology Bulletin*, 2017,  PMID 28191983 | Assessment 5 years to adulthood; meta-analysis of mental health problems; 1974-2003 | ≤1000 g BWT | International cohorts, all languages | Children born preterm showed increased risk compared to term controls for ADHD, internalizing and externalizing symptoms, conduct and oppositional disorders, ASD symptoms, social difficulties, depression, and anxiety. |
| Pierrat, *British Medical Journal*, 2017,  PMID 28814566 | Assessment at 2 years; Survival and development screen, neurosensory;  1997 vs. 2011 | 22-26 weeks GA | France, EPIPAGE and EPIPAGE-2 cohorts | No survivors at 22-23 weeks. Survival without moderate-or-severe neurosensory impairment in infants born at 25-26 weeks increased from 45% to 62% but did not change for infants born at 24 weeks, 29% to 26%. |
| Samuelsson, *Pediatrics*, 2017,  PMID 28642374 | Assessment at 10 to 15 years; behavior, emotions;  1992-1998 | 23-25 weeks GA | Sweden, two centers | Infants born at 23-25 weeks GA, compared to term controls, had significantly increased rates of ≥90^th^ percentile for ADHD scores (parents: 40% vs. 15%) and significantly increased frequency of abnormal behaviors, adjusted odds ratio 3 (both parents and teachers). |
| Synnes, *Archives of Disease in Childhood, 2017*  PMID 27758929 | Assessment at 21 month corrected GA; NDI;  2009-2011 | <29 weeks GA | Canada, Canadian Neonatal Follow-up Network | 37%, 29%, and 21% of survivors born at ≤23, 24, and 25 weeks, respectively, had significant NDI. 63%, 62%, and 52% had any NDI. |
| Younge, *New England Journal of Medicine*, 2017,  PMID 28199816 | Assessment at 18-22 months corrected GA; Survival and NDI;  2000-2003 vs. 2004-2007 vs. 2008-2011 | 22-24 weeks GA | USA, Neonatal Research Network | Survival without NDI increased from 16% in Epoch 1 to 20% in Epoch 3. <1% and 1% of infants born at 22 weeks survived without NDI in Epochs 1 and 3. 7% and 13% of infants born at 23 weeks survived without NDI in Epochs 1 and 3. |
| Adams-Chapman, *Pediatrics*, 2018,  PMID 29666163 | Assessment at 18-26 months corrected GA; NDI;  2011-2015 | ≤27 weeks GA | USA, Neonatal Research Network | Rate of moderate-or-severe NDI (BSID III <85) of survivors did not significantly change over time, 34% vs. 31%. |
| Burnett, *Pediatrics*, 2018, PMID 29196505 | Assessment at 8 years; executive functioning; 1991-1992 vs. 1997 vs. 2005 | <28 weeks GA  <1000 g BWT | Australia, Victoria cohorts | Significantly lower executive function scores in premature infants compared to controls in all 3 eras, most notable in the latest cohort. |
| Chang, *Journal of the Formosan Medical Association*, 2018, PMID 29454513 | Assessment at 24 months corrected GA; Survival and NDI; 2007-2011 | <27 weeks GA | Taiwan, Taiwan Premature Infant Developmental Collaborative Study Group | Survival rates were 8, 25, 47, 67, and 77% at 22 to 26 weeks GA, respectively. Rate of NDI in survivors were 75, 65, 50, 40, and 33% at 22 to 26 weeks GA, respectively. |
| Hirschberger, *Pediatric Neurology*, 2018,  PMID 29310907 | Assessment at 10 years; IQ + executive function, CP, autism, epilepsy;  2002-2004 | 23-27 weeks GA | USA, ELGAN cohort | 52% of survivors born at 23-24 weeks had ≥1 impairment in assessed areas; 26% had ≥2 impairments. |
| Inoue, *Pediatrics*, 2018, PMID 30446630 | Assessment at 3 years; NDI;  2003-2012 | ≤500 g BWT | Japan, Neonatal Research Network | No significant change in the mean NDI rate of 59% in survivors over 10 years. |
| Linsell, *Archives of Disease in Childhood*, 2018,  PMID 29146572 | Assessment at 6, 11 and 19 years; IQ; 1995 | <26 weeks GA | United Kingdom  and Ireland, EPICure cohort | No change in rates of impaired cognitive function from 6 through 19 years of age. Mean IQ scores of survivors born at <26 weeks averaged 25 points lower than control infants born at term (80 vs. 105). |
| Nakanishi, *Journal of Perinatology*, 2018,  PMID 29679045 | Assessment at 3 years; NDI;  2003-2012 | 22-24 weeks GA | Japan, Neonatal Research Network | No change in cognitive impairment rates (Kyoto Scale/BSID III <85) in survivors born at 22-24 weeks (35-40%). Significant decrease in rates of neurosensory impairments; non-linear decrease in CP from 22% to 18%. |
| Spittle, *Pediatrics*, 2018, PMID 29567814 | Assessment at 8 years; motor;  1991-1992 vs. 1997 vs. 2005 | <28 weeks GA  <1000 g BWT | Australia, Victoria cohorts | Non-CP motor impairment rate increasing across the 3 eras (23% to 26% to 37%). No change in rate of CP (11-12%). |
| Brumbaugh, *JAMA Pediatrics,* 2019  PMID 30907941 | Assessment at 18-26 months corrected GA; Survival and NDI;  2008-2015 | <400 g BWT | USA, Neonatal Research Network | 23% of actively treated infants survived to follow-up; 74% of survivors had moderate-or-severe NDI. |
| Crump, *JAMA*, 2019,  PMID 31638681 | Assessment at 18-43 years; Survival and adverse health conditions including mental health;  1973-1997 | 22-27 weeks GA | Sweden,  Swedish Birth Registry | 51% survived to 18 years. Significantly fewer preterm infants born at 22-27 weeks GA survived with no major comorbidities (22%) compared to infants born at 28-33 weeks (48%) or full-term (63%). |
| Ding, *Acta Paediatrica*, 2019,  PMID 30537197 | Assessment at 4-10 years; meta-analysis of NDI;  1995-2007 | 22-25 weeks GA | International cohorts, English language | Rates of moderate-or-severe NDI in survivors were 42%, 41%, 32%, and 23% at 22, 23, 24, and 25 weeks GA respectively. |
| Myrhaug, *Pediatrics*, 2019, PMID 30705140 | Assessment at 18-36 months corrected GA; meta-analysis of survival and development; publications 2000-2017 (birth years unclear) | 22-27 weeks GA | International cohorts; English, German, French, or a Scandinavian language | Moderate-or-severe NDI rates in survivors were 61%, 50%, 42%, and 33% at 22, 23, 24, and 25 weeks GA respectively. Risk of NDI was not different for children born in the more recent years. |
| Pittet-Metrailler, *Swiss Medical Weekly*, 2019, PMID 31154661 | Assessment at 5 years; Survival and NDI; 2006 | <30 weeks GA | Switzerland, national cohort | Survival rates of 0, 27, 66, 71 and 87% for live born infants at 23, 24, 25, 26 and 27 weeks GA, respectively. 21% of survivors had mild cognitive impairment; 3.5% severe cognitive impairment; 3% mild CP; 2.5% severe CP; <1% major hearing or visual impairment. |
| Rysavy, *Journal of Pediatrics*, 2019,  PMID 30738658 | Assessment at 18-22 months corrected GA; Survival and NDI;  2006-2011 | 22-26 weeks GA | USA, Neonatal Research Network | Survival without moderate-or-severe NDI was 9%, 16%, 31%, 45%, 59% at 22, 23, 24, 25, and 26 weeks. |
| Kaul, *Acta Paediatrica*, 2020, PMID 32945030 | Assessment at 6.5 years; NDI;  2004-2007 | 22-27 weeks GA | Sweden, Longitudinal study of Visuomotor capacity | 14% of survivors had CP; none had blindness or hearing loss requiring amplification. Relative risk for a full-scale IQ >1 SD and >2 SD below mean were significantly increased in survivors at 2.41 and 8.82, respectively, compared to controls. |
| Watkins, *Journal of Pediatrics,* 2020*,*  PMID 31606151 | Assessment at 18-22 months corrected GA; Survival and NDI;  2006-2015 | 22-25 weeks GA | USA, University of Iowa | 22-23 week survival 78%; 36% had moderate or severe NDI.  24-25 week survival 89%; 24% had moderate or severe NDI.  No NDI improvement trend in either GA group reported. |
| O’Reilly, *Pediatrics*, 2020, PMID 31924688 | Assessment at 19 years; IQ, other cognitive;  1995 | <26 weeks GA | United Kingdom and Ireland, EPICure cohort | 45% of survivors born at <26 weeks had major full-scale IQ >2 SDs below mean compared to 3% of survivors born at term. |
| Yates, *Pediatrics*, 2020, PMID 32276969 | Assessment at 13 years; mental health;  2001-2003 | <30 weeks GA  <1250 g BWT | Australia, Victorian Infant Brain Study | 27% of children born preterm met criteria for one or more disorders (ADHD, ASD, anxiety, mood) vs. 6% of children born at term. |
| Cheong, *JAMA Pediatrics*, 2021, PMID 34279561 | Assessment at 2 years; Survival and NDI;  1991-1992 vs. 1997 vs. 2005 vs. 2016-2017 | <28 weeks GA | Australia,  Victoria cohorts | Survival (73%) was significantly higher in 2016-2017 (vs 53 to 70% in previous eras). Major NDI rates (15-26% of survivors) have not changed across 4 eras. Rates of survival free of major NDI have increased significantly over time (62% in 2016-2017 vs 42% in 1991-1992). |
| Marlow, *Archives of Disease in Childhood*, 2021,  PMID 33504573 | Assessment at 11 years; NDI;  1995 vs. 2006 | <26 weeks GA | United Kingdom and Ireland, EPICure and EPICure2 cohorts | Moderate (32% vs. 21%) or severe (18% vs. 26%) NDI rates have not changed across 2 eras in survivors, EPICure vs. EPICure2. |
| Pierrat, *British Medical Journal*, 2021,  PMID 33910920 | Assessment at 5 years; NDI, behavioral; 2011 | 24-26 weeks GA | France, EPIPAGE-2 cohort | Moderate-or-severe NDI in 34%, 26%, and 28% of survivors born at 24, 25, and 26 weeks, respectively. Mild NDI in 36%, 41%, and 38%, respectively. No NDI in 31%, 34%, and 34%, respectively. |
| Wang, *Journal of Formosan Medical Association*, 2021, PMID 33478783 | Assessment at 2 years; NDI;  1995-2000 vs 2001-2006 vs 2007-2016 | 23-26 weeks GA | Taiwan, regional cohort | In terms of a yearly trend, survival without NDI increased for infants born at 25-26 weeks GA but not at 23-24 weeks GA (where ~50% of survivors had NDI). |
| Morsing, *Acta Paediatrica*, 2022, PMID 35318709 | Assessment at 2 to 13 years; NDI;  2007-2018 | <24 weeks GA | Sweden, national registries | 75% of 22 and 23 week survivors had significant neurodevelopmental disorders including a broad range of cognitive, motor, behavioral, and psychiatric conditions. |
| Bell, *JAMA,* 2022*,*  PMID 35040888 | Assessment at 22-26 months corrected GA; NDI;  2013-2016 | 22-26 weeks GA | USA, Neonatal Research Network | 21% had severe NDI, 29% moderate NDI, 49% no or mild NDI. |
| van Beek, *Archives of Disease in Childhood*, 2022  PMID 35236745 | Assessment at 2 years corrected GA; NDI; 2018-2020 | 24-26 weeks GA | Netherlands extreme prematurity cohort | Bayley-III cognitive scores >/= 85 were 81%, 70-84 16%, and <70 3%. Motor scores >/= 85 were 75%, 70-84 21%, and <70 4%. |
| Ricci, *Journal of Pediatrics*, 2022  PMID 35561804 | Assessment at 18-24 months corrected GA; NDI;  2009-2012 vs  2013-2016 | 22-28 weeks GA | Canada,  Canadian Neonatal Network | Death or significant composite NDI (Bayley III <70) was lower in the later era (30%) compared to the earlier era (32%). Moderate cognitive NDI (Bayley III <85) increased in the later era (16%) compared to the earlier era (13%). In 22-25 week infants, death or significant composite NDI trended improvement, but not at a significant level. |
| **Definitions and abbreviations**: ADHD – attention deficit disorder, ASD – autism spectrum disorder, BSID - Bayley Scales of Infant Development; BWT - birth weight; CP - cerebral palsy; DQ - developmental quotient; ELGAN – extremely low gestational age; EXPRESS - extremely preterm infant study in Sweden; GA - gestational age; g - grams; IQ - intelligence quotient; NDI - neurodevelopmental impairment which includes assessment of cognition, motor, vision, and hearing.  Neurosensory - includes motor, vision, hearing, not DQ or IQ.  **Notes**:   1. This table is the result of a systematic review, (see reference #48, Kaempf). It serves to provide the reader with a list of high-quality publications reporting on the range of neurodevelopmental outcomes of extremely premature infants. Readers can interrogate the methodologies and data further to understand the many complexities involved in these follow-up studies of children born extremely preterm. 2. One respective cohort may be reported in more than one publication. It can be difficult to comprehend the exact prevalence of the various neurodevelopmental outcomes in the individual children represented in population-based data. 3. We have shown the ***Principal Findings*** in either positive (“survival”, “free of NDI”) or negative (“death”, “have NDI”) framing. 4. Some of the reported data in ***Principal Findings*** is an outcome combination of “Death or NDI”. 5. In cohorts with term controls, the term control group often has an NDI rate of ~3%, considering low cognition, cerebral palsy, and visual/hearing impairment as NDI. 6. Most publications do not report on the array of behavioral, educational, and neuropsychiatric impairments. Few clarify what a “normal” child is. 7. Cohorts vary in level of quality, for example, size, attrition rates, statistical analysis used for not followed, and/or retrospective versus prospective methodology. 8. Not all studies use the same definitions for impairment classifications. “Mild” versus “Moderate” versus “Severe” NDI classifications are not 100% standardized, particularly when comparing American reports to other countries. See each reference for clarity and comparison. The current 2022 National Institute of Childhood Health and Human Development (NICHD) standard is the Bayley Scales of Infant Development III - mean score 100, 1 standard deviation 15. “Normal or Mild NDI” NDI is defined as >/= 85; Moderate NDI as 70-84; Severe NDI <70. 9. The term “significant NDI” applies only to each study’s calculated statistical significance (p-values and confidence Intervals). The neurodevelopmental assessments are used to provide objective metrics to assist comparisons of complex neurologic outcomes across centers and countries. | | | | |
